# Advances in Brain Tumor Biomarkers: From Molecular Profiling to Liquid Biopsy and AI-Driven Detection

**DOI:** 10.3390/cancers18111779

**Published:** 2026-05-29

**Authors:** Trang T. T. Nguyen, Lan N. Ðoàn, Evgenii Boriushkin, Christian E. Badr

**Affiliations:** 1 Ronald O. Perelman Department of Dermatology, Perlmutter Cancer Center, NYU Grossman School of Medicine, NYU Langone Health, New York, NY 10016, USA; 2 Department of Population Health, NYU Grossman School of Medicine, New York University, New York, NY 10016, USA; lan.doan@nyulangone.org; 3 Tropocan, Inc., New York, NY 11790, USA; eboriushkin@tropocan.com; 4 Department of Neurology, Massachusetts General Hospital, Neuroscience Program, Harvard Medical School, Boston, MA 02129, USA; badr.christian@mgh.harvard.edu

**Keywords:** glioma, biomarkers, liquid biopsy, artificial intelligence (AI), circulating tumor DNA (ctDNA), cerebrospinal fluid (CSF)

## Abstract

Brain tumors in older adults are difficult to diagnose and manage due to nonspecific symptoms, overlapping with neurodegenerative diseases such as dementia, and significant tumor heterogeneity. Although molecular markers such as IDH1/2 mutations, MGMT promoter methylation, TERT alterations, and 1p/19q co-deletion have improved glioma classification and prognostic assessment, current care still relies on invasive tissue biopsies, which limit longitudinal monitoring and may not fully capture tumor complexity because of sampling bias, assay variability, and limited accessibility. Liquid biopsy offers a promising alternative that enables the detection of tumor-derived DNA, RNA, proteins, and extracellular vesicles, supporting earlier diagnosis and real-time monitoring of disease progression and treatment response. However, liquid biopsy for brain tumors is not yet clinically definitive due to low biomarker abundance, lack of standardization, and limited validation, and therefore, it cannot replace tissue diagnosis. Ongoing research focuses on multi-analyte biomarker panels, improved assay standardization, and integration with imaging and tissue-based data. In parallel, artificial intelligence and machine learning are advancing the field by integrating multi-omics and radiomic data to enhance detection, classify tumors, and predict key molecular alterations, supporting the emerging framework of radiogenomics. Together, these developments are driving a shift toward more precise and dynamic approaches to brain tumor diagnosis and management, with relevance for improving outcomes in older adults with brain cancer.

## Simple Summary

Brain tumors in older adults are often difficult to detect early because symptoms can mimic normal aging or dementia, and diagnosis currently depends on imaging and, in some cases, tissue biopsy. These approaches also have limitations in capturing tumor heterogeneity and changes over time. Liquid biopsy has emerged as a promising complementary tool by analyzing blood or cerebrospinal fluid for tumor-derived signals, with the potential to support earlier detection and ongoing monitoring, although it is not yet sensitive or standardized enough to replace tissue biopsy. Artificial intelligence is further advancing this field by integrating molecular and imaging data to improve the detection and tracking of brain tumors. Together, these developments are moving the field toward earlier, more precise, and more adaptive brain tumor care.

## 1. Introduction

Brain tumors in older adults often present with nonspecific neurological symptoms, including memory loss, confusion, and personality changes, which can overlap with neurodegenerative conditions such as dementia or Alzheimer’s disease [1,2]. As a result, brain tumors are often not found early and may only be diagnosed after clear problems with brain function appear, when treatment options are fewer and outcomes are often worse [3,4]. Despite advances in our understanding of tumor biology, current diagnostic strategies remain heavily reliant on neuroimaging followed by surgical biopsy or resection for histopathological and molecular characterization [5]. While effective, these approaches are inherently invasive, episodic, and limited in their ability to capture spatial and temporal tumor heterogeneity or to support longitudinal disease monitoring [6]. This underscores a critical unmet need for sensitive, minimally invasive biomarkers that enable earlier detection and more dynamic assessment of disease progression.

Structural and demographic disparities may further influence diagnosis and outcomes in older adults. Structural disparities are systemic differences in access to healthcare resources and clinical trial enrollment. In the context of brain tumors, these disparities may delay diagnosis, reduce access to clinical trials, and limit the use of new diagnostic and treatment tools [7]. Older adults, as well as racial and ethnic minority populations, are often underrepresented in clinical and molecular trials, limiting the generalizability of novel therapies and access to quality care [8,9]. Together, these barriers further complicate pathways for timely diagnosis, treatment decision-making, clinical trial participation, and the delivery of quality care.

Aging and tumorigenesis share fundamental molecular hallmarks, including genomic instability, telomere dysfunction, epigenetic alterations, and chronic inflammation. Progressive telomere shortening contributes to cellular senescence and genomic instability, whereas aberrant telomere maintenance mechanisms are frequently co-opted by cancer cells to support uncontrolled proliferation [10]. In parallel, the accumulation of senescent cells and the senescence-associated secretory phenotype promote a pro-inflammatory microenvironment that can facilitate tumor initiation and progression [11,12,13]. Dysregulated autophagy, metabolic reprogramming, and persistent low-grade inflammation further disrupt tissue homeostasis [14]. Importantly, these interconnected processes not only drive disease pathogenesis but also generate measurable molecular alterations that may serve as candidate biomarkers for early cancer detection.

Current methods for diagnosing and tracking disease still have limitations. Imaging tools like Magnetic Resonance Imaging (MRI) and Computed Tomography (CT scans) do not always capture what is happening at the molecular level in real time, meaning early changes in the disease can be missed. Also, these scans can sometimes be misleading because treatment effects—such as pseudoprogression—may look like the disease is getting worse when it is not [15,16]. In this context, liquid biopsy has emerged as a promising approach for capturing tumor-derived material, including circulating tumor DNA, RNA, proteins, and extracellular vesicles, from biofluids such as blood and cerebrospinal fluid [17,18]. These approaches offer the potential for real-time, longitudinal monitoring of tumor dynamics and may more accurately reflect intratumoral heterogeneity. Concurrently, advances in artificial intelligence (AI) and machine learning are transforming biomarker discovery and clinical interpretation. By integrating high-dimensional, multi-omics datasets with clinical and radiographic data, AI-driven approaches can identify subtle molecular signatures, improve diagnostic accuracy, and enable more precise patient stratification [19,20,21,22,23]. In brain tumors, such integrative frameworks hold promise for distinguishing true progression from treatment-related changes and facilitating earlier detection.

In this review, we examine biomarkers for detecting adult brain tumors, including tissue-based, immunologic, chronic inflammatory, and procoagulant biomarkers. We also highlight the emerging role of liquid biopsy and AI-driven approaches in enabling earlier diagnosis and more precise, real-time disease monitoring. By integrating these biomarkers with multimodal patient data—including MRI, tissue biopsy, and liquid biopsy—AI-based frameworks can support key clinical applications across the care continuum, including early detection, monitoring of minimal residual disease (MRD), assessment of treatment response, and development of personalized therapeutic strategies.

## 2. Tissue-Based Molecular Biomarkers in Glioma

Tumor tissue analysis remains the gold standard for characterizing gliomas and guiding clinical management. Molecular profiling of surgical or biopsy specimens, including DNA sequencing, methylation analysis, and copy number assessment, allows identification of key biomarkers that inform diagnosis, prognosis, and treatment selection [24]. In accordance with the 2021 World Health Organization (WHO) classification, routine assessment of markers such as MGMT promoter methylation, IDH1/IDH2 mutations, TERT promoter mutations, and 1p/19q co-deletion provides critical information on tumor behavior and therapeutic responsiveness [25]. Beyond these established markers, gliomas harbor a complex landscape of genetic alterations, commonly affecting TP53, PTEN, CDKN2A, and EGFR, which contribute to tumor initiation, progression, and resistance mechanisms [26]. Understanding the interplay of these alterations is increasingly essential for precision medicine approaches in glioma care.

*O-6-methylguanine-DNA methyltransferase (MGMT) Promoter Methylation:* The *MGMT* gene encodes a DNA repair enzyme that removes alkyl adducts from the O6 position of guanine, thereby counteracting the cytotoxic effects of alkylating agents [27]. Promoter methylation silences MGMT expression and is present in approximately 40% of primary GBM and up to 75% of secondary GBM, frequently co-occurring with *TP53* mutations [15]. Clinically, MGMT promoter methylation predicts improved response to alkylating chemotherapies such as temozolomide, with median overall survival extending from 12.2 months in unmethylated tumors to 18.2 months in methylated tumors [28].

*IDH1/IDH2 Mutations:* Isocitrate dehydrogenase (IDH) enzymes catalyze the conversion of isocitrate to α-ketoglutarate while generating NADPH, which supports cellular redox homeostasis [29]. Recurrent heterozygous point mutations (e.g., *IDH1 R132H*) confer a neomorphic enzymatic activity that produces the oncometabolite D-2-hydroxyglutarate (D-2HG), leading to widespread epigenetic dysregulation through inhibition of DNA and histone demethylation [30,31]. *IDH* mutations are present in 73–85% of secondary GBM but are rare in primary GBM [32]. In gliomas, *IDH* mutations are associated with improved prognosis and enhanced responsiveness to therapy, and D-2HG levels may serve as a biomarker for monitoring treatment response [33,34].

*Telomerase Reverse Transcriptase (TERT) Promoter Mutations:* Telomerase activation is a hallmark of GBM, enabling cellular immortalization and genomic stability [10,11,35]. Hotspot mutations in the TERT promoter (C228T and C250T) are detected in 60–80% of GBM cases and are associated with poorer prognosis, particularly in primary GBM and in patients receiving incomplete resections or no temozolomide therapy [36,37].

*1p/19q Co-deletion:* The combined loss of chromosome arms 1p and 19q is a defining molecular hallmark of oligodendrogliomas and is strongly associated with favorable response to chemoradiotherapy and prolonged survival [25,38,39]. 1p/19q co-deletion is rare in GBM and is found in only about 2.5% to 5.7% of cases [40].

*TP53 Mutation:* The *TP53* gene, located on chromosome 17p13, is the most frequently mutated tumor suppressor in human cancer [41]. It encodes p53, a central regulator of DNA repair, cell cycle arrest, apoptosis, and senescence. In response to cellular stress, p53 accumulates to preserve genomic integrity or induce cell death. Mutations in *TP53* disrupt these protective mechanisms, allowing damaged cells to evade apoptosis and proliferate. More than 7000 *TP53* mutations have been identified, with over 70% consisting of missense variants clustered in the DNA-binding domain at hotspot residues (R175, G245, R249, R273, and R282) [42]. Next-generation sequencing (NGS) studies report *TP53* mutation frequencies of approximately 48% in colon adenocarcinoma, 36% in non-small cell lung carcinoma, and 28% in GBM [43]. In rarer subtypes such as gliosarcoma, *TP53* mutations occur in up to 70% of cases compared to 32% in GBM and are associated with poorer survival [44,45].

*Cyclin Dependent Kinase Inhibitor 2A (CDKN2A) Deletion: CDKN2A* encodes the tumor suppressor proteins p16 and p14, which regulate cell cycle progression and TP53 pathway activity [46]. It is frequently inactivated in cancer through deletion, mutation, or promoter methylation, contributing to tumor progression and metastasis [47]. A large meta-analysis demonstrated that *CDKN2A/B* deletions are significantly associated with reduced survival in both *IDH*-wildtype and *IDH*-mutant gliomas, with a stronger prognostic impact observed in *IDH*-mutant tumors [48,49,50]. Homozygous or hemizygous deletions of *CDKN2A* are commonly assessed using complementary molecular techniques.

*Phosphatase and Tensin Homolog (PTEN) Alteration: PTEN* is a tumor suppressor gene frequently inactivated in GBM, particularly in primary (IDH-wildtype) tumors. Mutations and loss of heterozygosity on chromosome 10q23 occur in 20–40% of cases and often co-occur with *EGFR* amplification [51,52]. Loss of PTEN function leads to activation of the PI3K/AKT signaling pathway, promoting tumor growth, survival, and therapeutic resistance. These alterations are associated with higher tumor grades, poorer prognosis, and shorter survival, and are considered late events in glioma progression.

*Epidermal Growth Factor Receptor (EGFR) Alteration: EGFR* alterations are among the most common molecular events in primary GBM, occurring in 40–60% of cases and contributing to aggressive tumor behavior. Amplification and overexpression are frequently observed, particularly in the classical molecular subtype, while *EGFRvIII* represents the most common mutant variant. EGFRvIII drives constitutive signaling, enhances tumor proliferation, and promotes immune evasion.

*Alpha-Thalassemia/Mental Retardation, X-linked (ATRX): ATRX* mutations are common in lower-grade astrocytoma and certain pediatric high-grade gliomas, frequently co-occurring with other key mutations, and they are associated with loss of ATRX expression that drives the alternative lengthening of telomeres pathway [53]. They occur in ~67–73% of grade II/III astrocytoma, ~57% of secondary glioblastomas, and 14–48% of pediatric high-grade gliomas and are rare in adult primary GBM, except in younger patients (~30%). Clinically, *ATRX* loss defines distinct molecular subtypes, is linked to specific co-mutation patterns (e.g., IDH, TP53, H3.3), and serves as a prognostic marker often associated with younger patient populations [54].

Collectively, these biomarkers are clinically validated and widely incorporated into the molecular classification, prognostic assessment, and therapeutic decision-making of gliomas. Biomarkers such as *MGMT* promoter methylation, *IDH1/IDH2* mutations, 1p/19q co-deletion, and TERT promoter mutations are now routinely evaluated in clinical practice and are integrated into the current World Health Organization classification system for CNS tumors. In addition, alterations involving *TP53*, *CDKN2A*, *PTEN*, *EGFR*, and *ATRX* provide important prognostic and biological insights, helping to stratify patients, predict treatment response, and guide precision medicine approaches in glioma management.

## 3. Liquid Biopsy

Conventional diagnostic approaches are limited by invasiveness, radiation exposure, high cost, and poor sensitivity for early-stage disease detection. In this context, liquid biopsy has emerged as a minimally invasive alternative that enables the analysis of tumor-derived biomarkers, including circulating tumor DNA (ctDNA), cell-free DNA (cfDNA), circulating tumor cells (CTCs), extracellular vesicles (EVs), and microRNAs, from body fluids such as blood, urine, and cerebrospinal fluid (CSF) [55,56,57,58,59,60]. Unlike traditional tissue biopsies that require surgical intervention, liquid biopsy relies on simple and repeatable sample collection, thereby reducing procedural risks while facilitating longitudinal monitoring and improved assessment of tumor heterogeneity at a lower cost [61].

The concept gained clinical traction in the past decade following the identification of circulating tumor cells in cancer patients. Liquid biopsy refers to the sampling of non-solid biological tissues to detect tumor cells or associated molecular alterations [58]. These tumor-derived biomarkers enter circulation through apoptosis, necrosis, or active secretion. Among them, CTCs provide valuable cellular and molecular insights into tumor biology and metastatic potential, although their low abundance limits sensitivity [62]. ctDNA, a tumor-derived fraction of cfDNA, reflects tumor-specific genomic alterations and serves as a powerful tool for mutation detection and treatment guidance, although it is often present at very low levels [63]. Extracellular vesicles, particularly exosomes, have emerged as a promising class due to their ability to carry tumor-derived DNA, RNA, proteins, and lipids within a protective lipid bilayer [59]. This structural stability preserves molecular cargo during circulation and enhances detectability. Notably, exosomes can traverse the blood–brain barrier, providing a more stable and enriched source of tumor-specific information compared to freely circulating analytes [64]. Increasing evidence suggests that exosomal RNA and protein signatures more accurately capture oncogenic alterations and intratumoral heterogeneity, supporting their potential for longitudinal disease monitoring and therapeutic stratification [65]. In addition, circulating microRNAs contribute to gene regulation and have demonstrated diagnostic and prognostic value, while aberrant DNA methylation patterns offer an additional epigenetic layer for cancer detection [60].

**Figure 1:**
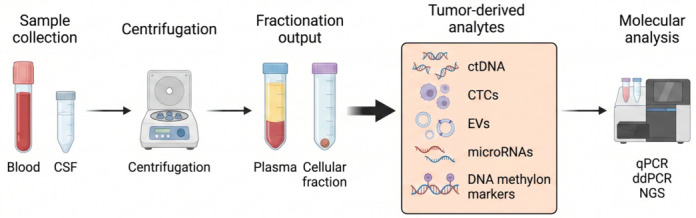
Workflow for isolation and analysis of tumor-derived analytes from liquid biopsy samples. Blood or cerebrospinal fluid (CSF) is collected and subjected to centrifugation to separate plasma from the cellular fraction in blood, or to isolate the cellular fraction from CSF. These fractions contain multiple tumor-derived components, including circulating tumor DNA (ctDNA), circulating tumor cells (CTCs), extracellular vesicles (EVs), microRNAs, and DNA methylation markers. The recovered analytes are then characterized using molecular assays such as quantitative PCR (qPCR), droplet digital PCR (ddPCR), and next-generation sequencing (NGS). Figure created with BioRender.

The workflow begins with collecting a blood or CSF sample, followed by centrifugation to isolate plasma or other components. Tumor-derived analytes including ctDNA, CTCs, EVs, microRNAs, and DNA methylation markers are then extracted and analyzed using molecular techniques such as quantitative PCR (qPCR), digital droplet PCR (ddPCR), and NGS [66] (Figure 1). Notably, the choice of biomarkers and analytical methods may vary between blood and CSF, reflecting differences in tumor shedding, analyte abundance, and the distinct biological properties of each fluid. For instance, an EGFR mutation in a brain tumor may be readily detectable in CSF, which is in closer contact with the CNS tumor, whereas the same mutation may be absent or present only at very low levels in blood because only limited amounts of tumor DNA enter the bloodstream.

In neuro-oncology, liquid biopsy primarily focuses on plasma and CSF [63,67]. Plasma-based approaches are minimally invasive but face significant challenges due to the blood–brain barrier, which limits the release of tumor DNA into circulation [68]. As a result, ctDNA detection rates in plasma for primary brain tumor patients are typically below 10%, lower than in other advanced cancers [69]. Highly targeted assays that detect specific known mutations have shown some success, and focused ultrasound has been tested to transiently open the blood–brain barrier and enhance ctDNA yield [70,71]. Despite these advances, low ctDNA abundance and the need for highly specific assays make plasma-based liquid biopsy technically challenging in brain tumors.

In contrast, CSF-based liquid biopsy has demonstrated superior performance, as ctDNA concentrations are significantly higher and more representative of the tumor’s molecular profile [72]. CSF typically contains fewer white blood cells, reducing background non-cancerous DNA and improving ctDNA detection accuracy [73]. The main limitation is its invasive collection, which restricts the frequency of sampling in routine practice [70].

Clinically, liquid biopsy shows promise across multiple applications. It can support diagnosis when tissue biopsy is not feasible, using sensitive techniques such as ddPCR and targeted NGS to detect tumor-specific alterations. It also enables detection of minimal residual disease (MRD) and early recurrence, often preceding radiographic progression [67,74]. Additionally, serial liquid biopsy sampling allows real-time monitoring of treatment response, with ctDNA dynamics correlating with disease burden, prognosis, and tumor evolution. Liquid biopsy can detect minute amounts of residual tumor that may predict relapses earlier than imaging. In pediatric medulloblastoma, monitoring tumor-associated copy-number changes in CSF served as a surrogate for MRD, with persistent MRD linked to higher progression risk [75]. Notably, molecular MRD often preceded radiographic evidence of relapse, suggesting that CSF-based monitoring could enable earlier therapeutic intervention. In one study of eighty-five glioma patients, tumor-derived DNA in CSF was detected in approximately 49% of cases, correlating with disease burden and prognosis. Genetic alterations in CSF closely mirrored tumor tissue, including key mutations such as *IDH1/IDH2* and 1p/19q codeletion, while also revealing tumor evolution over time [76]. Another study compared CSF collected near the tumor versus lumbar puncture, showing that tumor DNA is more abundant proximal to the tumor but still detectable in over 60% of lumbar puncture samples for key alterations (*IDH*, TERT promoter, *EGFR*, *CDKN2A/B*, *MGMT*), supporting tissue-independent molecular profiling [77].

Beyond genetic alterations, emerging evidence suggests that tumor-derived metabolic and stress-response signatures may represent an underexplored class of liquid biopsy biomarkers in glioma. Dysregulation of lipid metabolism, a hallmark of GBM, can influence membrane composition, redox balance, and DNA repair capacity, potentially influencing the profile of circulating tumor-derived analytes [78]. In particular, altered fatty acid desaturation and lipid saturation states may promote cellular stress responses linked to DNA damage signaling and cell death pathways. More broadly, recent work has highlighted functional links between metabolic stress and impaired homologous recombination, raising the possibility that metabolic vulnerabilities may be indirectly captured through circulating biomarkers [79]. Together, these observations support the idea that integrating metabolic and stress-response signatures with conventional genomic liquid biopsy approaches could improve sensitivity and provide deeper insight into tumor biology and treatment response.

Despite its considerable promise, brain tumor liquid biopsy still faces several unresolved challenges that limit its clinical implementation. Important gaps remain in our understanding of the fundamental biology of ctDNA, including how it is released, its temporal kinetics, and the ways treatment influences its detectability over time. Interpretation is further complicated by substantial inter-patient variability, which also hampers standardization across studies [80]. Practically, although CSF-based liquid biopsy yields a higher tumor signal, it requires invasive lumbar puncture and is therefore less suitable for frequent longitudinal monitoring. In contrast, plasma-based assays offer a minimally invasive alternative, but their sensitivity remains insufficient for reliable routine clinical use [81].

## 4. Age-Associated Immune Reprogramming Biomarkers

As we age, the immune system becomes less effective at detecting and eliminating abnormal cells, a process called immunosenescence [82]. Concurrently, chronic low-grade inflammation creates a tissue environment that can support tumor growth [83]. These age-related immune alterations weaken anti-tumor defenses, modify interactions between cancer cells and their surroundings, and influence responses to therapy. Understanding this interplay is crucial for developing tailored treatments for older adults, especially in aggressive cancers like GBM, where the immune environment strongly affects patient outcomes [84].

Recent research investigated how aging shapes the immune microenvironment in primary versus recurrent GBM. By analyzing patient survival, large-scale gene expression, ~89,000 single tumor cells, and mouse models, the study found that older age negatively impacts survival more strongly in primary tumors than in recurrent ones [85]. Notably, while tumors in older patients carry more genetic mutations, this alone does not explain their poorer outcomes. Instead, age-related changes in the surrounding immune environment appear to drive these differences. Primary tumors in older patients are dominated by microglia, whose activity is suppressed, whereas recurrent tumors show more therapy-influenced immune cell populations. In older primary GBM, intercellular signaling favors tumor growth and invasion, and the stress-response gene HSPB1 is elevated in microglia, correlating with worse survival [85]. These findings highlight distinct biological mechanisms between primary and recurrent tumors and suggest that targeting age-associated microglial alterations may improve outcomes for older GBM patients.

Building on this understanding of immune dysregulation in GBM, a phase I randomized clinical trial (NCT04201873) evaluates whether combining PD-1 blockade (pembrolizumab) with autologous tumor lysate-pulsed dendritic cell (ATL-DC) vaccination can enhance anti-tumor immunity in recurrent GBM (Table 1) [86]. The study uses a neoadjuvant and adjuvant design with extensive longitudinal immune monitoring across tumor tissue, peripheral blood, and imaging, enabling integrated assessment of T cell dynamics, TCR clonality, and gene expression programs. These multi-dimensional immune readouts are then correlated with clinical endpoints such as progression-free survival (PFS) and overall survival (OS), providing a framework to connect age-associated immune alterations with therapeutic response and resistance mechanisms in GBM.

Despite promising findings, several limitations remain in the study of age-associated immune reprogramming biomarkers in GBM. The tumor immune microenvironment is highly heterogeneous and influenced by factors such as treatment history, tumor stage, and patient variability, making it difficult to establish consistent biomarkers [87]. Many current findings are correlative and derived from small cohorts or preclinical models that may not fully reflect immune aging in humans. In addition, older adults are often underrepresented in clinical trials, limiting the clinical validation and broader application of these biomarkers for personalized GBM therapy.

## 5. Chronic Inflammatory and Procoagulant Biomarkers

In cancer care, circulating biomarkers can provide prognostic information beyond standard clinical assessments. In acute myeloid leukemia, for instance, patterns of cytokines—key immune signaling molecules—better predicted patient survival than traditional measures of overall health, identifying those at highest risk of poor outcomes [88,89]. Similar principles apply to brain cancers, where gliomas exist within a highly dynamic tumor microenvironment in which inflammation and coagulation interact to drive disease progression. Tumor cells and stromal populations, including microglia, macrophages, and endothelial cells, secrete pro-inflammatory cytokines such as interleukin-6 (IL-6), interleukin-1β (IL-1β), and tumor necrosis factor (TNF), which activate oncogenic pathways including JAK–STAT and NF-κB [90,91]. These pathways promote proliferation, angiogenesis, and immune evasion, while elevated cytokines (IL-6, IL-8) and chemokines (CCL2) in tumor tissue and cerebrospinal fluid reflect the intensity of tumor-associated inflammation [92,93].

Systemic studies support the prognostic relevance of these markers. A meta-analysis of thirty-one studies including 2587 glioma patients found that elevated blood levels of IL-6, IL-8, IL-17, TNF-α, TGF-β, and C-reactive protein (CRP) were associated with both increased risk of glioma development and poorer survival outcomes [94,95,96]. In a prospective cohort of 163 patients with meningioma or glioma, higher IL-6 levels correlated with worse discharge outcomes and increased mortality in high-grade glioma, whereas elevated high-sensitivity CRP (hsCRP) predicted higher mortality in meningioma but not short-term outcomes [97]. These findings suggest that IL-6 and hsCRP serve as complementary prognostic biomarkers, with IL-6 reflecting both short- and long-term outcomes in glioma and hsCRP indicating survival risk in meningioma. Importantly, these observations suggest that systemic inflammation is not merely a correlate of disease burden but may actively contribute to tumor progression and therapeutic resistance.

Beyond inflammatory mediators, coagulation biomarkers such as D-dimer have emerged as important predictors of prognosis in GBM [98]. Pre-treatment D-dimer levels, serum albumin, and the albumin-to-D-dimer ratio have been significantly associated with progression-free and overall survival. Elevated D-dimer reflects a tumor-driven hypercoagulable state, which increases thromboembolic risk and correlates with more aggressive tumor biology [99]. Mechanistically, pro-inflammatory cytokines upregulate tissue factor expression, amplifying coagulation pathways, while coagulation proteases such as thrombin activate inflammatory signaling through protease-activated receptors (PARs), creating a feed-forward loop that reinforces glioma progression [100,101].

Clinical trial NCT05133154 is a prospective observational study that evaluates longitudinal plasma and cerebrospinal fluid biomarkers in glioma patients to monitor disease evolution and treatment response over time (Table 1). The trial focuses on serial assessment of circulating tumor-derived components alongside systemic inflammatory markers, with the goal of correlating biomarker dynamics with imaging findings and clinical outcomes. Overall, it aims to improve early detection of progression and better distinguish true tumor progression from treatment-related effects.

Collectively, these data support a model in which inflammatory and procoagulant pathways converge to shape glioma biology at both local and systemic levels. Integrating circulating tumor DNA with cytokine and coagulation profiling provides a multidimensional approach for improving diagnosis, risk stratification, and longitudinal monitoring in patients with glioma.

A major limitation of chronic inflammatory and procoagulant biomarkers in glioma is their lack of specificity, as markers such as IL-6, CRP, and D-dimer can also be elevated by infections, autoimmune conditions, treatment effects, or other systemic inflammatory states [96,102]. Variability in patient populations, sample processing, and biomarker cutoff values further limits reproducibility across studies. In addition, many studies are retrospective and include small or heterogeneous cohorts, reducing clinical generalizability. Although these biomarkers show prognostic potential, larger prospective studies are still needed to validate their reliability and clinical utility in glioma management.

## 6. Clinical Trials in Liquid Biopsy for Diagnosis, Monitoring, and Precision Therapy

Recent clinical trials have increasingly demonstrated that liquid biopsy approaches, when integrated with molecular profiling, hold significant promise for improving glioma diagnosis, monitoring, and therapeutic personalization [76]. These studies utilize ctDNA, cell-free DNA (cfDNA), RNA biomarkers, and advanced imaging techniques to reduce reliance on invasive tissue biopsies while enabling more precise, real-time patient management.

Several clinical trials focus on non-invasive diagnostic strategies for GBM diagnosis and monitoring. The clinical trial NCT05964153 is a pilot interventional study evaluating the feasibility and diagnostic accuracy of ctDNA detection in patients with suspected gliomas, comparing blood-based molecular profiles with conventional tissue biopsy results (Table 2). In addition, the circTeloDIAG trial (NCT04931732) is a prospective observational study evaluating a blood-based biopsy for glioma diagnosis by targeting key biomarkers such as *IDH* mutations, TERT promoter mutations, and *ATRX* loss, with the goal of determining whether these markers can serve as reliable alternatives to tissue biopsy for early detection and disease monitoring (Table 2).

Other studies emphasize therapy monitoring and personalized treatment. The BRAIN MATRIX platform (NCT04274283) is a large-scale observational initiative enrolling approximately 1000 glioma patients and integrating genomic data from tumor tissue and plasma ctDNA to identify actionable alterations and guide targeted therapies (Table 2) [103]. The personalized ctDNA-guided trial (NCT05539339) explores serial ctDNA analysis from tumor in situ fluid following resection to detect early molecular relapses and guide individualized treatment decisions (Table 2). Similarly, the PLANET study (NCT05099068) collects sequential tumor biopsies and blood samples to monitor molecular and immunological tumor evolution and correlate these changes with treatment response and resistance (Table 2). Additional trials, including NCT05630664 and NCT05925218, investigate ctDNA, cfDNA, and circulating tumor cells as longitudinal biomarkers to track tumor burden, characterize tumor biology, and assess disease progression across different glioma subtypes (Table 2). Notably, the ExoGLIE study (NCT06116903) introduces an exosome-based liquid biopsy approach, evaluating whether NGS of exosomal nucleic acids can improve the detection of tumor-specific alterations compared with standard plasma-based assays, thereby offering a more sensitive method for monitoring tumor evolution in recurrent GBM (Table 2).

Emerging studies are also exploring innovative and adjunctive approaches to enhance liquid biopsy performance. The RESTORE LB trial (NCT07417774) evaluates blood biomarkers in GBM patients receiving chemoradiation combined with experimental oxygen therapy, investigating how tumor hypoxia influences treatment response and clinical outcomes (Table 3). Clinical trial NCT05383872 investigates MRI-guided focused ultrasound-mediated transient blood–brain barrier disruption as a strategy to enhance the release of ctDNA into the bloodstream, with the goal of improving the sensitivity and reliability of liquid biopsy detection (Table 3).

## 7. Integrating AI with Liquid Biopsy for Precision Detection of Brain and CNS Tumors

AI is rapidly reshaping the field of liquid biopsy, offering transformative opportunities to improve the detection, classification, and monitoring of cancers, particularly those that are difficult to access, such as gliomas [55,104,105]. By integrating machine learning (ML) and deep learning (DL) approaches, researchers are beginning to overcome longstanding limitations in liquid biopsy by detecting subtle yet biologically meaningful patterns within complex datasets, thereby enhancing sensitivity and expanding the range of potential applications [106,107]. One emerging example of this progress is the development of blood-based, AI-driven assays for brain tumor detection. At Memorial Sloan Kettering Cancer Center, researchers are designing sensors made from modified carbon nanotubes that capture blood proteins reflecting not only the tumor itself, but also the host immune response and tumor microenvironment [108,109]. AI algorithms then analyze these protein signatures to identify patterns, much like facial recognition systems that detect distinguishing features. In early studies, this approach demonstrated high accuracy—up to 98%, although validation in larger cohorts is still needed—in distinguishing tumor from non-tumor samples, with some ability to differentiate among tumor types. If further validated, such technology could enable earlier and less invasive detection of brain tumors through a simple blood draw [108].

Beyond mutation detection, AI is also revolutionizing the analysis of fragmentomics and methylomics in liquid biopsy [107]. Deep learning models, including convolutional neural networks, can interpret cfDNA fragmentation patterns and genome-wide methylation landscapes to accurately classify cancer types and predict tissue of origin—even in cases where tumor-specific mutations are unknown or highly heterogeneous [110,111]. Importantly, AI facilitates the integration of multi-omics data, including genomics, transcriptomics, and proteomics into unified models that can guide precision oncology strategies [69,70].

**Figure 2:**
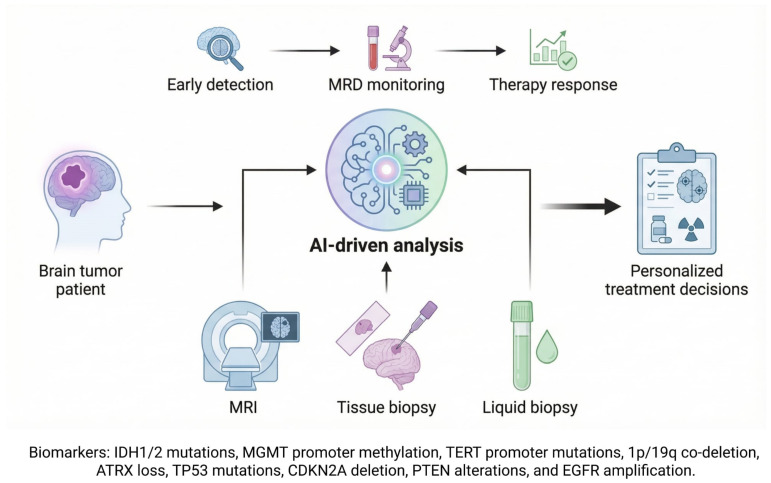
AI-integrated multimodal framework for precision brain tumor management. Clinical data from brain tumor patients—including MRI, tissue biopsy, and liquid biopsy—are harmonized within a centralized AI-driven analytical platform. This integrated framework enables comprehensive, high-resolution data interpretation to support critical clinical applications, including early detection, minimal residual disease (MRD) monitoring, and real-time assessment of therapeutic response. By synthesizing multimodal insights, the platform facilitates informed, personalized treatment decisions, advancing precision oncology and improving outcomes in brain tumor care. MRD: Minimal Residual Disease, IDH: isocitrate dehydrogenase, MGMT: O-6-methylguanine-DNA methyltransferase, TERT: Telomerase Reverse Transcriptase, ATRX: Alpha-Thalassemia/Mental Retardation, X-linked, CDKN2A: Cyclin Dependent Kinase Inhibitor 2A, PTEN: phosphatase and tensin homolog, EGFR: epidermal growth factor receptor. Image created with BioRender.

Despite these advances, several challenges must still be addressed before AI-driven liquid biopsy can be fully integrated into clinical practice. Robust model development depends on access to large, well-annotated datasets, as well as standardized protocols for sample collection, processing, and analysis. At the same time, biological constraints intrinsic to gliomas—notably the low abundance of ctDNA in blood due to the blood–brain barrier—continue to limit assay sensitivity [112,113]. Although CSF can provide a more informative source of tumor-derived material, its collection is invasive and therefore not always practical for longitudinal monitoring. In addition, the BBB and other compartment-specific biological factors lead to significant differences between blood and CSF in the abundance, molecular composition, and clinical interpretability of tumor-derived analytes. These biofluid-specific distinctions have implications not only for biomarker detection and assay design but also for the development, training, and validation of AI-based models, which may be influenced by distinct signal characteristics in each compartment. Beyond these technical and biological limitations, regulatory and translational barriers also remain: no liquid biopsy assays are currently FDA-approved for glioma, and AI-based diagnostics require rigorous external validation across independent cohorts. Finally, issues related to model interpretability and seamless clinical integration must be resolved to support safe, effective, and widespread adoption in routine care (Figure 2).

Several ongoing clinical trials are addressing key translational gaps across the glioma care continuum. For instance, clinical trial NCT05536024 investigates the integration of advanced MRI-based radiomics with blood-derived tumor biomarkers to noninvasively predict glioma molecular features before surgery (Table 4). By leveraging AI to decode imaging patterns and infer tumor aggressiveness and mutation status, this study directly addresses the need for multimodal preoperative characterization within a noninvasive framework [114]. In a complementary manner, clinical trial NCT07420543 evaluates a multimodal intraoperative approach that combines 5-aminolevulinic acid-induced fluorescence imaging with liquid biopsy biomarkers to improve real-time tumor visualization in GBM (Table 4). In addition to enhancing surgical precision, this strategy examines how biomarker signals reflect underlying tumor biology, with the broader aim of refining molecular characterization at the time of resection.

Clinical trial NCT06717295 is studying whether RNA patterns found in platelets, small blood components involved in clotting, can be used to detect cancer earlier and monitor how patients respond to treatment over time (Table 4). Using artificial intelligence, the study looks for specific RNA “signatures” that can help tell the difference between people with cancer and those without it and track how these signals change during therapy. By focusing on platelets instead of only tumor DNA in the blood, this approach expands liquid biopsy methods and may make cancer detection more sensitive and useful in the clinic.

Scientists are increasingly using an approach called “convergence science,” which brings together biology with fields like physics, chemistry, and computer modeling. This combined approach is helping researchers better understand how the immune system works at a detailed level. New computational tools, such as TAMAVAQ (NCT07077616), can simulate how the immune system might respond to vaccines, help identify better treatment strategies, and support doctors in making more informed clinical decisions (Table 4). Together, these approaches can work alongside AI-based liquid biopsies and advanced imaging to give a more complete picture of how tumors develop, how the immune system responds, and how treatments are working over time (Figure 2).

Overall, this reflects a broader shift in cancer research toward combining different technologies—such as blood-based tests, medical imaging, and computer analysis—to overcome the limitations of any single method (Figure 2). The biggest limitations of AI in liquid biopsy, imaging, and biopsy are not just algorithm performance. They are data quality, bias, generalizability, validation, interpretability, workflow fit, and proof of real clinical benefit. AI can be powerful in all three areas, but safe and effective use requires rigorous validation across diverse real-world settings.

## 8. Future Direction and Conclusions

Brain tumors in older adults pose distinct diagnostic and therapeutic challenges. Their clinical presentation often overlaps with that of neurodegenerative disorders, while tumor heterogeneity and the limitations of conventional imaging and tissue-based diagnostics can further hinder accurate diagnosis. These challenges are particularly relevant when considering biopsy, as advanced age may increase procedural risk through greater tissue fragility, delayed healing, and a higher likelihood of complications such as bleeding, bruising, and prolonged recovery. Consequently, the decision to pursue biopsy in older adults requires careful weighing of diagnostic benefit against procedural risk, especially in patients who are frail or have limited life expectancy. This limitation is especially consequential because, although molecular classification of gliomas—including biomarkers such as IDH1/2 mutations, MGMT promoter methylation, TERT promoter alterations, and 1p/19q co-deletion—has substantially improved prognostication and enabled more personalized treatment strategies, its reliance on invasive tissue sampling continues to constrain longitudinal monitoring and real-time assessment of tumor evolution [38]. A key limitation of current biomarker strategies in glioma is their inability to fully capture the complexity of tumor biology. Significant intra-tumoral heterogeneity means that distinct cellular subclones within the same tumor can harbor different genetic and epigenetic profiles, reducing the representativeness of a single biopsy specimen [115]. This contributes to sampling bias, where tissue obtained from one region may not reflect the broader molecular landscape of the tumor, particularly in spatially and temporally evolving disease. In addition, assay variability across platforms and laboratories can introduce inconsistencies in biomarker detection and interpretation, complicating clinical decision-making. Practical considerations further limit implementation; for example, high costs and unequal access to advanced molecular testing technologies can restrict their availability, especially in community or resource-limited settings [25]. Collectively, these challenges underscore the need for more robust, standardized, and accessible approaches to biomarker development and integration in neuro-oncology.

Liquid biopsy has emerged as a promising minimally invasive strategy to help overcome many of these limitations, particularly in central nervous system malignancies, where CSF provides a highly informative source of tumor-derived material. CSF-derived circulating tumor DNA can support earlier detection, monitoring of minimal residual disease, and real-time assessment of treatment response, and it may more accurately capture the molecular landscape of glioblastoma than peripheral blood [116]. Despite substantial technological and analytical progress, however, liquid biopsy is not yet ready to replace surgical tissue diagnosis in GBM, and no FDA-approved assays currently exist specifically for this disease. Several challenges continue to limit its clinical adoption, including intratumoral heterogeneity, limited analytical sensitivity (particularly in early-stage or low-shedding tumors), the lack of standardized protocols, and insufficient validation across diverse patient populations. Overcoming these barriers will require large, prospective, multicenter studies conducted with harmonized methodologies, as well as rigorous efforts to reduce test–retest variability and improve reproducibility.

In addition, systemic biomarkers, including inflammatory cytokines and coagulation factors, may further enhance prognostic accuracy while providing important insight into glioma biology. This is particularly relevant in the context of age-related immune remodeling, which highlights the pivotal role of the tumor microenvironment in shaping disease progression and therapeutic response, especially in elderly patients. Building on these observations, future research should prioritize the development of multiparametric biomarker panels rather than relying on single analytes, as integrated molecular signatures are more likely to provide greater biological robustness and stronger clinical relevance (Figure 2). In parallel, expanding the spectrum of detectable genetic and epigenetic alterations in both blood- and cerebrospinal fluid-based assays will be essential to improve sensitivity, accessibility, and scalability. To facilitate clinical translation, rigorous comparative studies against established standards, including tissue biopsy and advanced imaging, are needed to better define utility in practice. Equally important will be careful consideration of cost-effectiveness and feasibility across diverse healthcare systems, particularly in resource-limited settings, to support equitable implementation. In this regard, recent advances in AI and machine learning further strengthen the field’s potential by enabling the sensitive detection of low-abundance signals, integrating complex multi-omics datasets with advanced imaging modalities, and supporting predictive modeling of disease trajectories [117,118] (Figure 2).

In conclusion, improving care for older adults with brain tumors will depend on biomarker strategies that are accurate, minimally invasive, and clinically accessible. While tissue-based molecular profiling remains indispensable, its limitations in elderly patients and in capturing dynamic tumor heterogeneity highlight the growing importance of liquid biopsy and integrated systemic biomarkers. Continued standardization, prospective validation, and incorporation of multiparametric and AI-enabled approaches will be essential to translate these advances into equitable, real-world neuro-oncology practice.

## Figures and Tables

**Table 1:** Clinical trials of immune and cytokine biomarkers in glioma.

Study Name	Clinical Trial	Study Period	Phase	Overview
Pembrolizumab and a Vaccine (ATL-DC) for the Treatment of Surgically Accessible Recurrent Glioblastoma	NCT04201873	8 January 2020–1 August 2027	Phase 1	The study investigates how biomarkers relate to clinical outcomes and safety in recurrent GBM patients treated with pembrolizumab and ATL-DC vaccine. It examines correlations between TIL density and IFN-gamma signatures with treatment response, evaluates PFS6, PFS, and OS using RANO and iRANO criteria, explores T cell expansions in tumors and blood, and assesses whether MRI changes reflect tumor and immune responses [84].
Liquid Biopsy in Low-grade Glioma Patients (GLIOLIPSY)	NCT05133154	15 December 2021–1 July 2025	Not Applicable	Utility of multiple blood-based biomarkers in DLGG patients for disease diagnosis and monitoring.

**Table 2:** Clinical trials on liquid biopsy for glioma diagnosis and monitoring.

Study Name	Clinical Trial	Study Period	Phase	Overview
Analysis of Circulating DNA in Blood Samples of Glioma-affected Patients	NCT05964153	31 January 2023–31 January 2024	Not Applicable	This study evaluates a blood-based liquid biopsy for glioma diagnosis using ctDNA detected by digital PCR. Results are validated against tissue biopsies, with the goal of developing a minimally invasive, accurate, and faster diagnostic approach.
The circTeloDIAG: Liquid Biopsy for Glioma Tumor	NCT04931732	4 November 2021–November 2026	Observational	A multi-marker liquid biopsy (ctDNA) approach targeting *IDH* mutations, TERT mutations, and *ATRX*-associated signatures in blood for non-invasive glioma detection and monitoring.
Tessa Jowell BRAIN MATRIX—Platform Study	NCT04274283	24 November 2020–February 2028	Observational	The study aims to enable rapid, accurate molecular diagnosis of brain tumors by building a UK-wide clinical network, supporting faster access to targeted, genetics-driven clinical trials [103].
Personalized Trial in ctDNA-level-relapse Glioblastoma	NCT05539339	1 December 2022–1 June 2025	Not Applicable	This pilot trial collects tumor in situ fluid (TISF) from GBM patients to analyze ctDNA for early detection of relapse.
Profiling Program of Cancer Patients with Sequential Tumor and Liquid Biopsies (PLANET)	NCT05099068	16 November 2021—15 September 2025	Not Applicable	Multi-cohort study aims to analyze tumors and liquid biopsies over time in advanced cancer patients to understand molecular profiles and adaptive mechanisms. It seeks to predict treatment response or resistance and guides therapeutic options through a molecular tumor board.
Liquid Biopsy in High-grade Gliomas and Meningiomas (SOPRANO)	NCT05630664	1 October 2022–30 September 2025	Observational	This project explores plasma cell-free DNA as a marker to predict treatment response and survival in high-grade glioma and meningioma patients.
Circulating Tumor DNA Collection from Patients with High Grade Gliomas (m-ctDNA)	NCT05925218	2 September 2022–30 September 2027	Observational	The study aims to develop a sensitive liquid biopsy for primary brain tumors, enabling non-surgical monitoring of tumor DNA in the bloodstream to track changes in tumor biology and improve outcomes for high-grade gliomas.
Clinical Relevance of Detecting Molecular Abnormalities in Glial Tumor Exosomes (ExoGLIE)	NCT06116903	11 April 2024–15 December 2025	Not Applicable	Evaluating whether tumor-derived exosomes in blood provide more sensitive and comprehensive molecular profiling than standard circulating tumor DNA-based liquid biopsy using NGS.

**Table 3:** Emerging clinical approaches to improve liquid biopsy detection.

Study Name	Clinical Trial	Study Period	Phase	Overview
Liquid Biopsy in Glioblastoma Treated with Chemoradiation and an Oxygen Therapeutic (RESTORE LB)	NCT07417774	8 December 2025–December 2030	Observational	This trial aims to identify biomarkers associated with tumor hypoxia, distinguish pseudoprogression from true progression, and correlate these biomarkers with clinical outcomes.
Blood–Brain Barrier Disruption for Liquid Biopsy in Subjects with Glioblastoma Brain Tumors	NCT05383872	8 August 2022–5 March 2025	Not Applicable	A pivotal study to evaluate the safety and effectiveness of exablate model 4000 using microbubble resonators to temporarily mediate blood–brain barrier disruption for liquid biopsy in subjects with GBM brain tumors

**Table 4:** Clinical trials of AI- and liquid biopsy-based platforms for early detection and disease monitoring.

Study Name	Clinical Trial	Study Period	Phase	Overview
Combing a Deep Learning-Based Radiomics with Liquid Biopsy for Preoperative and Non-invasive Diagnosis of Glioma	NCT05536024	1 May 2022–30 August 2023	Observational	This registry develops and validates a multi-task deep learning model for non-invasive glioma diagnosis and combines radiomics with liquid biopsy to improve diagnostic accuracy and clinical decision-making [112].
Clinical Significance of Liquid Biopsy in Brain Tumor Patients: a 5-ALA Guided Approach (FLUO-LB)	NCT07420543	18 January 2017–May 2027	Observational	This prospective observational study leverages 5-ALA-induced fluorescence to enhance plasma liquid biopsy for diagnosis, post-treatment monitoring, and prognosis.
The CCANED-CIPHER Study: Early Cancer Detection and Treatment Response Monitoring Using AI-Based Platelet and Immune Cell Transcriptomic Profiling	NCT06717295	20 December 2025–1 August 2028	Observational	This multi-center study aims to develop an AI-based blood test analyzing platelet and immune cell biomarkers to detect cancer early and monitor treatment responses non-invasively.
Clinical Study for the Safety and Therapeutic Efficacy of the AI-QMMM Designed TamavaqTM Personalised Vaccine in Patients with Newly Diagnosed Glioma.	NCT07077616	1 July 2025–1 December 2029	Early Phase 1	This study evaluates safety in GBM patients over 28 weeks by systematically tracking adverse events (graded by CTCAE), physiological toxicity, and quality of life alongside MRI and laboratory monitoring. It also integrates advanced imaging, liquid biopsy biomarkers, and AI-driven analyses to assess tumor response and long-term treatment efficacy.

## Data Availability

No new data were created or analyzed in this study. Data sharing is not applicable.

## Abbreviations

The following abbreviations are used in this manuscript:

AI	Artificial Intelligence
ATRX	Alpha-Thalassemia/Mental Retardation, X-linked
CDKN2A	Cyclin Dependent Kinase Inhibitor 2A
cfDNA	cell-free DNA
CSF	Cerebrospinal Fluid
ctDNA	Circulating Tumor DNA
CT	Computed Tomography
CTCs	Circulating Tumor Cells
EGFR	Epidermal Growth Factor Receptor
EVs	Extracellular Vesicles
GBM	Glioblastoma
IDH	Isocitrate Dehydrogenase
MGMT	O-6-Methylguanine-DNA Methyltransferase
MRI	Magnetic Resonance Imaging
NGS	Next-Generation Sequencing
OS	Overall Survival
PFS	Progression-free Survival
PTEN	Phosphatase and Tensin Homolog
TERT	Telomerase Reverse Transcriptase
